# LEVERAGING THE NDUFS4-/- MOUSE AS A PLATFORM FOR TESTING LONGEVITY INTERVENTIONS

**DOI:** 10.1093/geroni/igac059.2663

**Published:** 2022-12-20

**Authors:** Alessandro Bitto, Cara Tobey, Ayush Sharma, Anthony Grillo, Matt Kaeberlein

**Affiliations:** University of Washington, Seattle, Washington, United States; University of Washington, Seattle, Washington, United States; University of Washington, Seattle, Washington, United States; University of Washington, Seattle, Washington, United States; University of Washington, Seattle, Washington, United States

## Abstract

Mitochondrial dysfunction is one of the hallmarks of biological aging, as well as the driving factor for mitochondrial diseases. Up to 30% of mitochondrial disorders are due to mutations affecting the activity of Complex I in the electron transport chain. Loss of the Complex I subunit Ndufs4 recapitulates symptoms of Leigh Syndrome, a pediatric mitochondrial disease, in mouse. Ndufs4-/- mice suffer developmental delays, early onset of neurological symptoms and extremely reduced lifespan. Several studies have now shown that Ndufs4-/- mice are exquisitely responsive to treatments and interventions of interest in the biology of aging, such as rapamycin, NAD+ precursors, reduced oxygen tension, alpha-keto-glutarate precursors, and the antidiabetic drug acarbose. These results point to common mechanisms underlying both aging and mitochondrial disorders. To put this hypothesis to the test, we show that Ndufs4-/- mice are responsive to a wide range of longevity interventions previously tested in worms, mice, and by the National Institute on Aging’s Intervention Testing Program. These observations support the hypothesis that mitochondrial and metabolic dysfunction induced by Complex I deficiency may be a key component of biological aging as well as mitochondrial disease. Furthermore, we propose that the Ndufs4-/- mice provide an affordable testing ground for candidate longevity interventions.

